# *Arthonia ulleungdoensis*, a New Lichenized Fungus from Ulleung Island, South Korea

**DOI:** 10.3390/microorganisms7070205

**Published:** 2019-07-19

**Authors:** Beeyoung Gun Lee, Jae-Seoun Hur

**Affiliations:** 1 Baekdudaegan National Arboretum, Bonghwa 36209, Korea; 2 Korean Lichen Research Institute, Sunchon National University, Suncheon 57922, Korea

**Keywords:** Arthoniaceae, biodiversity, corticolous, phylogeny, taxonomy

## Abstract

*Arthonia ulleungdoensis* Lee & Hur is described as a new lichen species from South Korea. The new species is distinguishable from *Arthonia ruana* A. Massal. by its large, rounded and non-punctiform apothecia, taller apothecial section, asci with fewer spores, and larger and permanently colorless spores. Molecular analyses employing mitochondrial small subunit (mtSSU) and RNA polymerase subunit II (RPB2) sequences strongly support *Arthonia ulleungdoensis* as a distinct species in the genus *Arthonia*. Overall, 22 *Arthonia* species are currently recorded in South Korea. A surrogate key is provided to assist in the identification of all 10 taxa of *Arthonia*/*Arthothelium* with muriform spores in Northeast Asia.

## 1. Introduction

The genus *Arthonia* is one of the least explored species in lichen taxonomy although the genus is comprised of about five hundred species worldwide [1]. One of the main reasons for this challenging genus is its ‘paraphyletic origin’. The relationship between *Arthonia* and related genera is unclear, and many *Arthonia* species have synonyms for *Arthothelium* or other genera. Molecular phylogeny also showed that *Arthonia* species are arranged over genera and even families [2]. Another reason for *Arthonia* is the ‘unstable structure’ of the species. Many species in *Arthonia* are highly pioneering. They disperse rapidly but become sterile with a deformed structure after spore discharge and dispersal in a short term. Therefore, although *Arthonia* species are encountered in the field, they are useless for analysis in many cases by the old, unfilled and barren ascomata with no ascus and spore. The third reason for the demanding genus is a ‘poor description’ of previous references as old descriptions are insufficient to explain specific characteristics of microlichens in anatomy, such as genus *Arthonia* particularly. The deficient depiction in the past is due mainly to the poor quality of microscopes, and still discourages a comparison with other species/specimens.

Although the genus *Arthonia* received much less attention in Asia compared to Europe and America, several Asian countries have actively studied *Arthonia*. In particular, in Asia, India has explored the genus the most since 2000 and dynamic studies such as local detection, new species/records discovery, diversity assessment and ethnolichenological survey for *Arthonia* were reported [3,4,5,6]. Turkey disclosed new species and records and reported other *Arthonia* species which were detected locally [7,8,9]. South Korea intently studied *Arthonia* since 2013 and many new species and records, including lichenized and lichenicolous fungi, were discovered [10,11,12]. For other countries, some new species/records were announced from Japan and three new records of foliicolous *Arthonia* species were reported recently from China [13,14,15].

**Figure 1:**
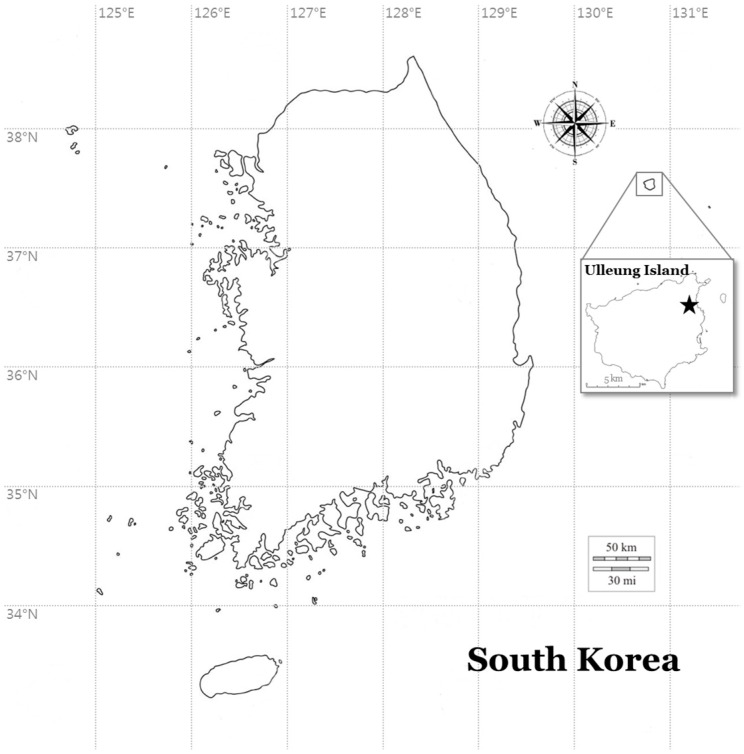
Specific collection site (black star mark) for the new species *Arthonia ulleungdoensis*.

This study aimed to describe a new lichenized fungus in the genus *Arthonia*. A concentrated field study was achieved in Ulleung Island and Pohang, which are an eastern island and seashore region of South Korea, during the summer of 2017 (Figure 1) and 30 specimens of *Arthonia* were collected. *Arthonia ulleungdoensis* was identified as a new species from Ulleung Island and this discovery revolves around 22 species in the genus *Arthonia* from South Korea, including a report on 12 new species/records of Korean *Arthonia* in 2016 [12]. All specimens were deposited in the herbarium of the Korean Lichen Research Institute (KoLRI). 

## 2. Materials and Methods

### 2.1. Morphological and Chemical Analyses

Hand-cut sections were prepared with a razor blade and examined under a stereomicroscope (Nikon SMZ645; Nikon, Tokyo, Japan) and a compound microscope (Nikon Eclipse E200; Nikon, Tokyo, Japan) and imaged using a software program (AxioVison Release 4.8.2; Carl Zeiss, Jena, Germany) and an Axiocam ERc 5s camera (Carl Zeiss, Jena, Germany) mounted on a Zeiss scope A1 microscope (Carl Zeiss, Jena, Germany). The ascospores were investigated at 1000× magnification in water. The length and width of the ascospores were measured and the range of spore sizes was shown with averages, standard deviation, and number of measured spores. Thin-layer chromatography (TLC) was performed using solvent systems A and C according to standard methods [16]. 

### 2.2. Isolation, DNA Extraction, Amplification, and Sequencing

Hand-cut sections of ascomata or thallus from all collected specimens were prepared for DNA isolation and DNA was extracted in line with the manufacturer’s instructions (Macherey-Nagel, Düren, Germany). Two-way PCR amplification for the mitochondrial small subunit (mtSSU) and RNA polymerase subunit II (RPB2) genes was achieved using Bioneer’s AccuPower PCR Premix (Bioneer, Daejeon, Korea) in 20-μL tubes and primers, mrSSU1 and mrSSU3R [17] and fRPB2-7cF and fRPB2-11aR [18], respectively. The PCR thermal cycling parameters used were previously described by Ekman [19]. Sequencing was accomplished by the genomic research company GenoTech (Daejeon, Korea).

### 2.3. Phylogenetic Analysis

All mtSSU and RPB2 sequences were aligned and edited manually using ClustalW in Bioedit (V7.2.5; Carlsbad, CA). The bootstrap values were obtained in RAxML GUI 1.5 beta (Heidelberg, Germany) [20] using the maximum likelihood method with a rapid bootstrap with 1000 bootstrap replications and GTR GAMMA for the substitution matrix. The posterior probabilities were obtained in BEAUti 1.8.0 and BEAST 1.8.0 [21] using the HKY (Hasegawa, Kishino and Yano) method for the substitution model, empirical base frequencies, gamma for the site heterogeneity model, four categories for gamma, and a 1,000,000 Markov chain Monte Carlo chain length with a 10,000-echo state screening and 200 log parameters. Then, the best tree was constructed in TreeAnnotator 1.8.0 [22] with a burn-in of 100, no posterior probability limit, a maximum clade credibility tree for the target tree type, and median node heights. All trees were displayed in FigTree 1.4.2 [23] and edited in Microsoft Paint.

## 3. Results and Discussion

**Figure 2:**
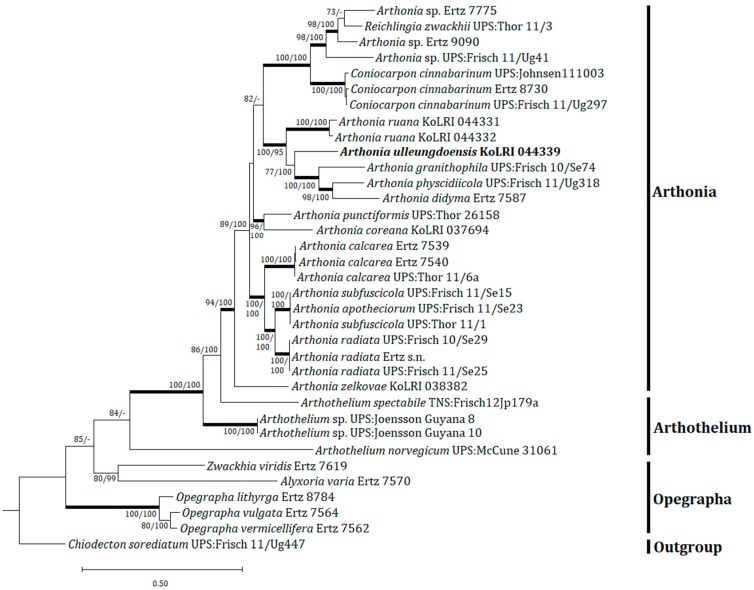
Phylogenetic relationships among the closely related species in the genera *Arthonia* and *Arthothelium* based on a maximum likelihood analysis of the concatenated dataset of all mitochondrial small subunit (mtSSU) and RNA polymerase subunit II (RPB2) sequences. The tree was rooted with a *Chiodecton sorediatum* sequence. Maximum likelihood bootstrap values ≥70% and posterior probabilities ≥95% are shown above internal branches. Branches with bootstrap values ≥90% are shown in bold. The new species *Arthonia ulleungdoensis* is presented in bold, and all species names are followed by the voucher information. Reference Table 1 provides the species related to the specific GenBank accession numbers and voucher information.

A concatenated tree was produced from two different loci, which involved overall 70 sequences (35 sequences for each mtSSU and RPB2) (Table 1). This concatenation employing mtSSU and RPB2 sequences allows a more comprehensive classification on diverse and comparable *Arthonia*/*Arthothelium* taxa to show the position of the new species in molecular phylogeny. The integrated tree shows that the new species is classified in the genus *Arthonia* (Figure 2). *Arthonia ulleungdoensis* is positioned in a highly supported clade with *Arthonia didyma* Körb., *Arthonia granitophila* Tr.Fr., *Arthonia physcidiicola* Frisch & G. Thor, and *Arthonia ruana*, represented by a bootstrap value of 100 and a posterior probability of 95 for the branch. The new species is separately located in the group without any closely positioned taxa. All *Arthothelium* taxa including *Arthothelium norvegicum* Coppins & Tønsberg, *Arthothelium spectabile* A. Massal., and *Arthothelium* sp. are situated quite far from the new species in the tree although those taxa are similar to the new species mainly by the spore morphology (i.e., muriform spores). *Arthonia ulleungdoensis* exhibits important characteristics of the genus *Arthonia*, particularly A. sect. *Arthonia*, such as maculate ascomata, olive-brown ascomatal pigments, persistently colorless spores, and photophilous [24], although the epihymenium is brownish without a distinct olive color for the new species. Regarding the above analyses and characteristics for the new species, *Arthonia ulleungdoensis* is identified as a unique species in both morphology and molecular phylogeny, being positioned in the genus *Arthonia*.

### New Species

**Figure 3:**
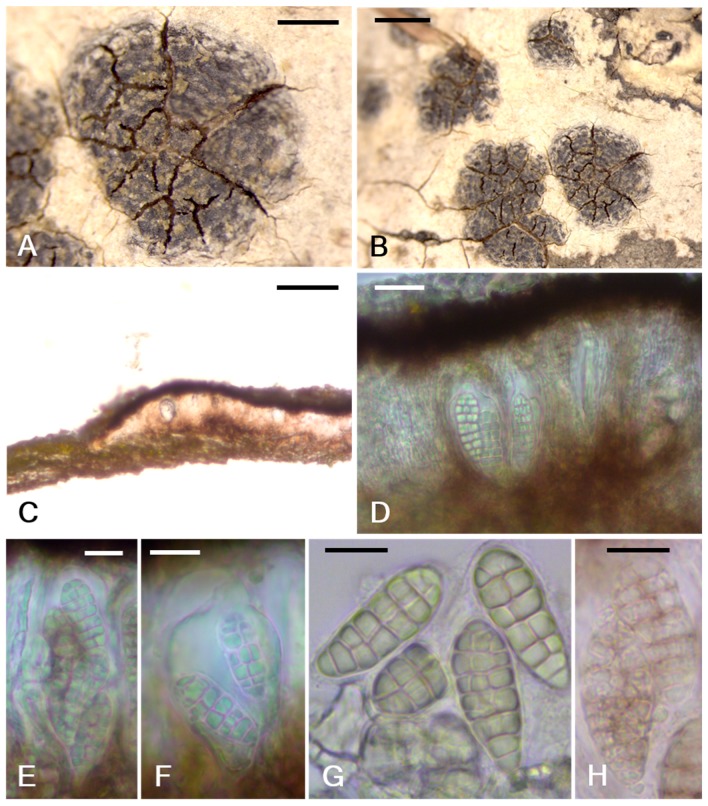
*Arthonia ulleungdoensis*. (**A**,**B**) Habitus with rounded and erumpent apothecia; (**C**) adnate apothecial section; (**D**) asci and ascospores in an apothecial section; (**E**) 6-spored ascus; (**F**) 2-spored ascus; (**G**) ascospores; (**H**) old ascospores with dark septa (scale bars: A = 1 mm; B = 0.5 mm, C = 100 μm, D to F = 20 μm, G and H = 10 μm).

*Arthonia ulleungdoensis* B.G.Lee & J.-S.Hur sp. nov. (Figure 3)

No.: MB823220

Type: South Korea, Gyeongsangbuk-do, Ulleung-gun, Ulleung-eup, Ulleung forest trail, 37° 31.20′ N; 130° 54.29′ E, 300 m alt., on *Acer takesimense* Nakai, 31 May 2017, B.G.Lee 170658 (holotype: KoLRI 044339!).

Thallus corticolous, crustose, hypophloedal, white to gray, without bleaching; epinecral layer 15–18 μm; medulla indistinct; photobiont trentepohlioid, forming a distinct algal layer, parallel to the substratum, between the epinecral layer and the brown bark layer or under apothecia, often in top layer of the brown bark, 45–54 μm thick, cells irregular, globose to angular, single or in chains, 4.5–6 × 6–9 μm, looking somewhat shrunken and supposed to be a little larger originally. Apothecia rounded, erumpent, black, epruinose, with or without epinecral bark layer on the surface of apothecia, 0.79–2.63 × 0.72–1.97 mm (length $\bar{x}$ = 1.56, SD = 0.73, width $\bar{x}$ = 1.32, SD = 0.51, *n* = 6); bark layer hyaline, 10–15 μm; apothecial section 100–130 μm thick; epihymenium brown to dark brown, 15–20 μm high; hymenium hyaline to light brown, 50–60 μm high, oil droplets near base of hymenium or in hypothecium; hypothecium brown to dirty brown, 25–35 μm high; paraphysoids septate, anticlinally arranged, 1–1.5 μm wide, somewhat branched at tips; tips swollen and pigmented, 1.5–2 μm wide. Asci wide to narrow clavate, 2-, 4- or 6-spored, 32–49 × 20–31 μm (*n* = 5); ascospores hyaline, muriform, 4- to 8-transverse and 0- to 3-longitudinal septa, without a gelatinous sheath, old spores with dark septum, 20.5–31 × 9–13 μm (length $\bar{x}$ = 25.9, SD = 2.81, width $\bar{x}$ = 11.4, SD = 2.60, *n* = 28). Pycnidia not detected.

Chemistry: K+ olive epihymenium and hypothecium, I+ light blue hymenium, and KI−. No substance detected by TLC (Thin Layer Chromatography).

Distribution and ecology: This species occur on the bark of *Acer takesimense*. It is currently known by a specimen in the type collection (Ulleung Island, South Korea). 

Etymology: This species epithet is named for the collection locality, South Korea.

Notes: *Arthonia ulleungdoensis* can be confused with *Arthonia ruana* in having black and epruinose apothecia, brown epihymenium, hyaline to light brown hymenium, brown hypothecium, K+ olive green reaction on an apothecial section, and the presence of a trentepohlioid alga [25]. However, the new species is distinguishable from the latter by larger, rounded and non-punctiform apothecia (0.7–2.6 mm *vs*. up to 1.6 mm), a taller apothecial section (100–130 μm *vs*. 70–95 μm), taller hymenium (50–60 μm *vs*. 35–50 μm), 2- to 6-spored asci (*vs*. 8-spored asci), and larger spores (20.5–31 × 9–13 μm *vs*. 15–26 × 7–10.5 μm) without changing their colors when mature (*vs*. brown old spores) [25]. The new species is similar to *Arthothelium norvegicum* in having a white thallus, black and rounded apothecia without pruina, brownish epihymenium and hypothecium, colorless hymenium, K+ greenish apothecial section reaction, muriform spores, and the presence of a trentepohlioid alga [25]. However, the new species differs from the latter by two to four times larger apothecia (0.7–2.6 mm *vs*. 0.3–0.6 mm diam.), smaller spores (20.5–31 × 9–13 μm *vs*. 29–36 × 12–15 μm), and colorless spores when mature (*vs*. brown old spores) [25]. The new species can be compared with *Arthothelium fecundum* Zahlbr. in having black, rounded and epruinose apothecia, dark-colored epihymenium and hypothecium, permanently colorless spores, similar ascospore size (20.5–31 × 9–13 μm *vs*. 25–27 × 9–11 μm), and the presence of a trentepohlioid alga [26]. However, the new species is distinctive from the latter by larger apothecia (0.7–2.6 mm *vs*. up to 0.7 mm diam.), immersed thallus (*vs*. superficial thallus), no prothallus (*vs*. brown-black prothallus), 2- to 6-spored asci (*vs*. 8-spored asci), and I+ light blue hymenium (*vs*. I+ reddish hymenium) reaction [26].

Key to the species in *Arthonia*/*Arthothelium* with muriform spores from Northeast Asia (10 taxa): This key includes all *Arthonia*/*Arthothelium* species with muriform spores in Korea (4), Japan (4) and China (3). However, only one species, *Arthothelium japonicum*, is excluded from the key because its ascomata represent a perithecioid type [27] and may not be an *Arthonia*/ *Arthothelium* species.

1. Photobiont *Protococcus* ………………………………………………………….*Arthothelium collosporum*

- Photobiont absent or *Trentepohlia* ………………………………………………………………………… 2

2. Hypothecium dark-colored ………………………………………………………………………………. 3

- Hypothecium colorless or pale brownish ………………………………………………………………... 5

3. Ascospores over 10 transversely-septate ……………………………………….*Arthothelium feduncum*

- Ascospores less than 10 transversely-septate …………………………………………………………… 4

4. Ascospores permanently colorless but with dark septum when mature, 20.5–31 × 9–13 μm, 2–6-spored asci ……………………………………………………………………………*Arthonia ulleungdoensis*

- Ascospores brownish when mature, 15–26 × 7–10 um, 8-spored asci

……………………………………………………………………….Arthonia ruana (Arthothelium dispersum)

5. Ascospores up to 10 transversely-septate ……………………………………..*Arthothelium punctatum*

- Ascospores less than 10 transversely-septate …………………………………………………………… 6

6. Thallus usually delimited by a brown line ………………………………………*Arthothelium spectabile*

- Thallus effuse without a delimited line ………………………………………………………………….. 7

7. Ascospores smaller, 16–20 × 7 μm …………………………………………….. *Arthothelium pertenerum*

- Ascospores larger, over 20 × 10 μm ………………………………………………………………………. 8

8. Ascospores 19–27 × 10–15 μm, photobiont not seen, hypothecium colorless

…………………………………………………………………………………….*Arthothelium scandinavicum*

- Ascospores 28–36 × 12–14 μm, photobiont *Trentepohlia*, hypothecium colorless or pale brownish ……………………………………………………………………*Arthothelium scandinavicum* var. *japonicum*

## Figures and Tables

**Table 1:** Species list and DNA sequence information employed for phylogenetic analysis.

	Species	mtSSU	RPB2	Voucher
1	*Alyxoria varia*	EU704075	EU704039	Ertz 7570
2	*Arthonia apotheciorum*	KJ850970	KJ851148	UPS:Frisch 11/Se23
3	*Arthonia calcarea*	EU704064	EU704028	Ertz 7539
4	*Arthonia calcarea*	EU704065	EU704029	Ertz 7540
5	*Arthonia calcarea*	KJ850974	KJ851105	UPS:Thor 11/6a
6	*Arthonia coreana*	KX913665	KX913668	KoLRI 037694
7	*Arthonia didyma*	EU704047	EU704010	Ertz 7587
8	*Arthonia granitophila*	KJ850981	KJ851107	UPS:Frisch 10/Se74
9	*Arthonia physcidiicola*	KF707646	KF707657	UPS:Frisch 11/Ug318
10	*Arthonia punctiformis*	KJ850973	KJ851113	UPS:Thor 26158
11	*Arthonia radiata*	EU704048	EU704011	Ertz s.n.
12	*Arthonia radiata*	KJ850968	KJ851108	UPS:Frisch 10/Se29
13	*Arthonia radiata*	KJ850969	KJ851109	UPS:Frisch 11/Se25
**14**	***Arthonia ruana***	**MG495135**	**MG589416**	**KoLRI 044331**
**15**	***Arthonia ruana***	**MG495136**	**MG589417**	**KoLRI 044332**
16	*Arthonia subfuscicola*	KJ850971	KJ851110	UPS:Thor 11/1
17	*Arthonia subfuscicola*	KJ850972	KJ851111	UPS:Thor 11/Se15
**18**	***Arthonia ulleungdoensis***	**MG495138**	**MG589418**	**KoLRI 044339**
19	*Arthonia zelkovae*	KX913667	KX913669	KoLRI 038382
20	*Arthothelium norvegicum*	KJ851003	KJ851114	UPS:McCune 31061
21	*Arthothelium spectabile*	KP870144	KP870160	TNS:Frisch12Jp179a
22	*Chiodecton sorediatum*	KF707648	KF707661	UPS:Frisch 11/Ug447
23	*Coniocarpon cinnabarinum*	KJ850977	KJ851104	UPS:Frisch 11/Ug297
24	*Coniocarpon cinnabarinum*	KJ850976	KJ851103	UPS:Johnsen 111003
25	*Coniocarpon cinnabarinum*	EU704046	EU704009	Ertz 8730
26	*Opegrapha lithyrga*	EU704068	EU704032	Ertz 8784
27	*Opegrapha vermicellifera*	EU704077	EU704041	Ertz 7562
28	*Opegrapha vulgata*	EU704080	EU704044	Ertz 7564
29	*Reichlingia zwackhii*	KF707652	KF707662	UPS:Thor 11/3
30	*Zwackhia viridis*	EU704078	EU704042	Ertz 7619
31	*Arthonia* sp.	EU704049	EU704012	Ertz 7775
32	*Arthonia* sp.	EU704050	EU704013	Ertz 9090
33	*Arthonia* sp.	KJ851025	KJ851100	UPS:Frisch 11/Ug41
34	*Arthothelium* sp.	KJ850958	KJ851094	UPS:Joensson Guyana 8
35	*Arthothelium* sp.	KJ850957	KJ851095	UPS:Joensson Guyana 10
	**Overall**	**35**	**35**	

DNA sequences for the new species and *Arthonia ruana* (in bold) were generated in this study. All others were obtained from GenBank. The species names are followed by GenBank accession numbers and voucher information. mtSSU, mitochondrial small subunit; RPB2, RNA polymerase subunit II; Voucher, voucher information.
